# Exploring a parent-focused physical literacy intervention for early childhood: a pragmatic controlled trial of the PLAYshop

**DOI:** 10.1186/s12889-022-13048-5

**Published:** 2022-04-05

**Authors:** Cassandra Lane, Patti-Jean Naylor, Madison Predy, Mette Kurtzhals, Ryan E. Rhodes, Kayla Morton, Stephen Hunter, Valerie Carson

**Affiliations:** 1 Hunter New England Population Health, Hunter New England Area Health Service, Newcastle, Australia; 2 School of Medicine and Public Health, The University of Newcastle, Newcastle, NSW Australia; 3 School of Exercise Science, Physical and Health Education, University of Victoria, Victoria, British Columbia Canada; 4 Faculty of Kinesiology, Sport, and Recreation, University of Alberta, 8840 114 St., Van Vliet Complex, University Hall, Edmonton, Alberta T6G 2H9 Canada; 5 Center for Clinical Research and Prevention, Frederiksberg Hospital DK, Frederiksberg, Denmark

**Keywords:** Physical literacy, Parents, Early childhood, Physical activity, At-home, Implementation

## Abstract

**Background:**

Parents play a key role in young children’s physical activity and physical literacy development. Little research has explored parent-focused interventions to improve young children’s physical literacy. We examined if a theory-based, feasible physical literacy training workshop (PLAYshop) for parents could improve their physical literacy knowledge and confidence and improve parenting practices related to facilitating the physical literacy development of their preschool-aged child (3-5 years). The secondary objective was to explore implementation facilitators and barriers.

**Methods:**

We conducted a pragmatic controlled trial in two Canadian cities (Edmonton and Victoria) from November 2019 – March 2020. A total of 143/151 parents were eligible and assigned to intervention (*n* = 71) or control group (*n* = 72). The PLAYshop included: (i) a 75-min in-person workshop with interactive activities and physical literacy educational messages, (ii) educational materials, (iii) an equipment pack, and (iv) two post-workshop booster emails. Surveys measured parents’ knowledge and confidence at baseline and follow-up. Application of PLAYshop concepts and implementation facilitators and barriers were explored with interviews of parents and workshop leaders. Repeated measures ANOVAs and thematic analyses were completed.

**Results:**

Parents’ knowledge and confidence improved significantly over time; intervention group changes were significantly greater than control group changes (*p* < 0.001; ɳ^2^ = .32). Parents applied PLAYshop concepts at-home, including child-led play, making activities fun, and promoting child manipulative and locomotor skills. Time was a key parental implementation barrier. Program implementation issues varied by context (location and participants).

**Conclusions:**

PLAYshop participation changed parents’ physical literacy knowledge and confidence and physical literacy enhancing play with their children. Implementation feasibility was high. The findings from this real-world trial highlight an efficacious and scalable intervention that warrants further testing.

**Trial registration:**

ClinicalTrials.gov: NCT04394312. Registered 19/05/2020.

**Supplementary Information:**

The online version contains supplementary material available at 10.1186/s12889-022-13048-5.

## Background

Physical activity in early childhood, the first 5 years of life, is associated with direct health benefits including improved motor and cognitive development as well as psychosocial and cardiometabolic health [1]. Unfortunately, many children internationally fail to meet physical activity guidelines [2]. Concomitantly, evidence suggests that children are lacking some of the foundational elements (i.e., movement competence, motivation, confidence, knowledge and/or understanding) that are necessary to engage in physical activity [3–5]. These elements are part of a comprehensive and holistic construct called physical literacy [6].

The understandings of physical literacy vary internationally, however common themes include philosophical underpinnings, core elements (motivation, confidence, physical competence, knowledge and understanding), and a life course perspective [7]. This is reflected in the International Physical Literacy Association (IPLA) definition of physical literacy, informed by Whitehead [8], as “the motivation, confidence, physical competence, knowledge and understanding to value and take responsibility for engagement in physical activities for life” [6]. Physical literacy incorporates many of the critical determinants of behavioral action from established theoretical paradigms (e.g., cognitive and humanistic) in health psychology (see Rhodes et al. [9] for an overview of the most relevant theories), similar to other meta-theoretical frameworks used in implementation science such as the Behaviour Change Wheel [10, 11]). Increasing research supports the targeting of physical literacy to address the childhood physical inactivity crisis. As a result, it is gaining attention from governments and organizations worldwide [12, 13]. Physical literacy is mutually inclusive of physical activity [14, 15], however it is underscored by its integration of various influences on physically active participation, including physical capabilities (e.g., Fundamental Movement Skills [FMS]) as well as affective (e.g., self-esteem and motivation) and cognitive elements (e.g., knowledge and understanding of movement and active play) [7, 15].

Although physical literacy is a life course concept, early childhood represents a significant window of opportunity for its development. First, theories of development [16, 17] and prior studies [18–20] suggest that younger children have high confidence in their ability to perform physical movements despite their actual motor competence. Positive self-perceptions are important for the physical activity trajectories of children during this time as they will be more likely to engage in active play pursuits, thus developing their physical capabilities, and affective and cognitive elements of physical literacy that support successive participation [16, 17]. A recent meta-analysis of 12 longitudinal studies found a positive association between FMS and physical activity in early childhood [21]. Actual motor competence and perceptions of motor competence are associated during childhood and thus mutually supportive [22]. Second, basic motor skill movements (e.g., striking, basic throwing, simple kicking, running and jumping) are developed during early childhood [23]. These are essential for learning more complex movements later in life [24], therefore children’s physical capabilities are an important target during this crucial time period. Lastly, physical activity throughout early childhood establishes positive patterns and routines that support participation in physical activity throughout the life course [16, 17, 25, 26].

To date, only a small number of studies have explored programs that promote physical literacy in early childhood. A 2021 systematic review of physical literacy interventions identified only four that targeted children under the age of five; all of which focused exclusively on childcare centers [27]. While these are important settings for intervention, several governing bodies –including the World Health Organization (WHO) [13] and Canada’s Sport for Life [23]– have highlighted the importance of meaningful engagement of parents in supporting young children on their physical literacy journey. Such endorsements are based on a growing body of research that highlights parents’ strong influence on their children’s physical activity-related behaviors [5, 28–30]. In a 2020 systematic review of the correlates of parental support for child physical activity [30], it was recommended that future interventions focus on “cognitive-behavioral approaches” of parental support for children’s physical activity as this had the highest evidence of association. Therefore, improving parents’ knowledge, confidence and play practices to encourage and facilitate their child’s physical literacy development offers considerable potential. Although several early childhood physical activity interventions have addressed and/or integrated parents – for example, ‘Healthy Dads Healthy Kids’ [31, 32] and the mother-daughter ‘MADE4Life’ program [33] – physical literacy was not addressed explicitly or comprehensively with little to no focus on parents’ cognitive-behavioral skills.

Drawing on the current evidence base and the noticeable gaps, we developed a brief, multi-strategy, theory-based physical literacy intervention (the PLAYshop) to influence parents' facilitation of their child’s physical literacy development. A recent one-group pretest-posttest study of parents with children aged 3-8 years revealed the feasibility of the PLAYshop [34]. Parents’ self-reported knowledge and confidence had significantly increased (*p* < 0.05) following participation and 95.6% intended to engage with their child in suggested activities. The majority of parents (81.8%) found the program very to extremely useful and acceptable, and 95% were satisfied or extremely satisfied with both the workshop content and delivery. This small-scale study provided valuable insight, however its methodological limitations, including lack of a control group and small sample size, restricted any conclusions of intervention efficacy. The following paper describes the subsequent controlled trial that was undertaken using more rigorous methods and with a larger sample of parents.

This study aimed to evaluate the preliminary efficacy of a theory-based, feasible and potentially scalable physical literacy intervention for parents of preschool-aged children (aged 3-5 years). The specific objectives are detailed below.

1. The primary objective was twofold: a) To determine if the PLAYshop increases parents’ knowledge and confidence. Based on previous evidence [30, 34, 35] and behavior change theories [10, 36], we hypothesized that parents in the intervention group will have a larger increase in levels of knowledge and confidence in regards to engaging in meaningful play with their preschool child(ren) than parents in the control group. b) To explore whether the PLAYshop improves parenting practices related to facilitating their child’s physical literacy development. We hypothesized that parents will report satisfaction with the implementation and change parenting practices related to physical activity at 2-month follow-up.
2. The secondary objective was to explore implementation facilitators and barriers.
3. The tertiary objective was to examine intervention effects on theoretical mechanisms of change (beliefs, perceived barriers, outcome expectations and perceived availability of resources). No specific hypotheses were developed.

## Methods

This study is reported in accordance with the TREND statement for reporting nonrandomized/quasi-experimental evaluations of behavioral and public health interventions [37]. It was retrospectively registered with the clinical trials registry maintained by the National Library of Medicine at the National Institutes of Health (ClinicalTrials.gov identifier NCT04394312).

### Study design and setting

**Fig. 1:**
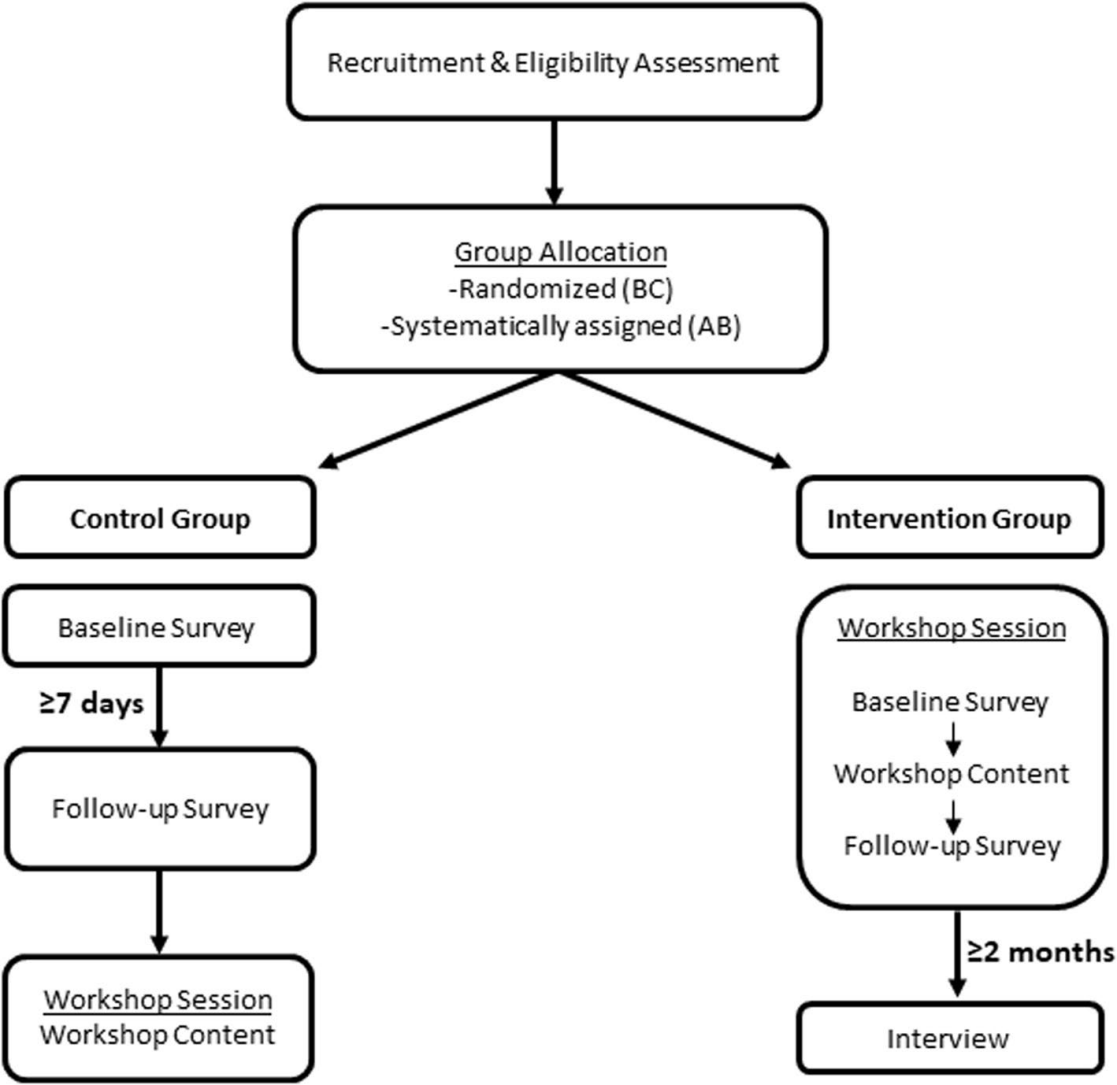
Study procedures for intervention and control groups

We used a type 1 effectiveness-implementation hybrid research design [38] to explore the preliminary efficacy of the PLAYshop and determine its potential for real world use. A two-arm controlled trial was conducted in the Canadian cities of Edmonton, Alberta (AB) and Victoria, British Columbia (BC) from November 2019 to March 2020. Figure 1 outlines the study procedures used for intervention and control groups. PLAYshop workshops were scheduled in advance at recreation centers and other community sites throughout the 4-month study period. The method used to allocate participants to either intervention or control group differed by region: BC participants were randomly assigned using a computer-generated 1:1 sequence and parents were provided with two choices for intervention workshop times; AB participants were systematically assigned using an alternating sequence as they enrolled and were provided with a list of workshop dates to choose from. Each approach was considered most feasible within that particular jurisdiction to balance groups and prevent wait-times that were anticipated to otherwise impact workshop attendance rates. To facilitate recruitment retention and the ethical treatment of control group participants, the control group in each cohort of recruits was booked in a delayed workshop and completed both pre- and post-test measures concurrent with the intervention participants who received the workshop in between (see Fig. 1).

Participants were blinded to group assignment however allocation concealment was not performed for those delivering the intervention due to limited resources (i.e., staff). To ensure intervention fidelity researchers were trained and delivered the PLAYshop using a pre-described program plan for the 75-min with added time points for each key message and related activities (i.e., fidelity checklist). Quantitative data (study objective 1a and 3) and qualitative data (study objective 1b and 2) were collected from participants and workshop leaders. Ethics approval was obtained from the University of Victoria (16-444) and University of Alberta (00093764). Written informed consent was received from participating parents and workshop leaders. All workshops from mid-March 2020 onwards were cancelled due to the COVID-19 pandemic, which halted recruitment and quantitative data collection. Workshops were not scheduled to resume due to public health restrictions and projected pandemic timelines.

### Participants and recruitment

Parent participants were recruited during the study period using informational posters placed in community recreation centers, daycare centers, and preschools (AB & BC); Facebook and Twitter posts (AB & BC); posters attached within a local electronic newsletter (AB); and posters emailed to parent playgroups (BC). Eligible parents had to live within a workshop delivery area and have at least one child aged 3-5 years. Parents and siblings outside the age range from the same family were welcome to co-participate, however data were collected from only one parent (self-selection). The initial sample size was set at a total of 100 parents (50 control and 50 intervention), providing an estimated 0.80 power for a medium to large effect size with alpha set at 0.05 for a t-test between two independent means. Workshop leaders (all five; members of the research team) were additionally recruited to address study objective 2.

### Intervention

A description of the PLAYshop development including theoretical underpinnings and a detailed logic model has been published elsewhere [34]. Briefly, the PLAYshop aimed to build parents’ knowledge and confidence to assist their child to develop physical literacy and acquire physical activity through play. Bandura’s social cognitive theory [36] broadly informed intervention design. The Behaviour Change Wheel [10, 11] was used to map the factors (capabilities, opportunities, and motivation of behavior [COM-B]) to identified barriers of parents’ target behavior. Potential implementation strategies were identified and assessed in terms of their affordability, practicality, effectiveness, cost-effectiveness, acceptability, side-effects/safety and equity [11], and with consideration of scalability (including adaptation to fit other contexts) from the onset [39, 40]. Table 1 is an extension from the feasibility study [34], describing the selected implementation strategies and the specific behavior change technique(s) used to address factors hypothesized to influence parents’ adoption of target behaviors. Strategy 3 (the provision of material resources) and strategy 4 (follow-up support) were added following the feasibility study to enhance contact time.

**Table 1:** Description of PLAYshop strategies mapped to the relevant COM-B [29] factors and behavior change techniques

Implementation strategy	Intervention function	Barriers and enablers addressed (COM-B [11])	Behavior change technique employed	Detailed description
**1. Conduct educational training**	-Education -Training -Modelling -Enablement -Persuasion	Parent knowledge and confidence (psychological capability).	-information about physical literacy and positive outcomes for child -instruction on how to perform the behavior(s) with equipment or common household items -demonstration of the behavior(s) -practice of the behavior(s) -problem solving -identification of self (parent) as role model to child -verbal persuasion about capability -principles of and ideas for modifications to support the parent in meeting the child’s needs in terms of current ability and motivations	A 75-min workshop for parents, delivered in an accessible community site (e.g., school, sport club or recreation center) by two leaders^a^ with a background in physical literacy. Parents are introduced to the core concepts of physical literacy (motivation, confidence, physical competence) via education, group discussion, and active participation in FMS-based activities. Parents are provided with modifications to perform activities ‘at home’.
		Parent perceived ability to implement change (reflective motivation).		
		Lack of available resources and/or time to engage in purposeful play with child (physical opportunity).		
		Lack of prioritizing child’s physical literacy (reflective motivation).		
**2. Distribute educational resources**	-Education -Enablement	Parent knowledge and abilities (physical and psychological capability).	-information about physical literacy, physical activity and positive outcomes for child -problem solving -messages about addressing multiple development goals through physical play (e.g., numeracy by counting with each ‘jump’)	Parents receive several resources at workshop conclusion: Canada’s 24-h movement guidelines for the early years, a booklet with various activity ideas, and a one-page physical literacy information handout.
		Lack of available resources and/or time to engage in purposeful play with child (physical opportunity).		
**3. Provide material resources** ^b^	-Environmental restructuring	Parent resources to engage in purposeful play with child (physical and social opportunity).	-addition of material resources in the at-home environment	Parents receive a ‘goody-bag’ with various content (e.g., a ball, scarves, bean bags and ribbons) at workshop conclusion to support child development of physical literacy at-home.
**4. Provide follow-up support** ^b^	-Education -Enablement	Parent knowledge, abilities and confidence to use workshop learnings (physical and psychological capability, and reflective motivation).	-social support -verbal persuasion about capability -provision of ongoing resources and support	Research staff send parents a follow-up email at week three and six inclusive of key workshop messages (e.g., rationale and importance of physical literacy); support and encouragement; and an opportunity for parents to provide feedback and/or seek further support.

^a^A co-leader was added after the feasibility study to assist with child management during the workshop

^b^New strategy, added after the feasibility study to enhance follow-up contact

*Abbreviations*: *COM-B* Capability-Opportunity-Motivation Behaviour, *FMS* Fundamental movement skills

The core component of the PLAYshop was a 75-min in-person workshop during which trained leaders provided parents and their child with physical literacy education and experiential learning (strategy 1). The workshop template and content were developed by a member of the research team (PJN) in collaboration with a local BC physical literacy agency (Pacific Institute of Sport Excellence [PISE]) using a combination of the best available evidence and first-hand experience. The active play activities were based on resources and training developed for preschool-aged children by experts in physical education and early years childcare [23, 41, 42] and based on formative qualitative research with childcare providers. Prior to refinement for evaluative research, the PLAYshop underwent an iterative process of feasibility testing for physical literacy content, adult behavior change techniques and delivery format. The resulting template was used to provide structure and maintain fidelity to the core concepts of physical literacy across settings.

The workshop targeted parent’s knowledge, confidence, competence and motivation to engage in purposeful play activities and games that developed their child’s physical literacy. The workshop exposed them and their child to a full range of playful activities (*n* = 19 + 12 adaptations) to support the development of FMS (8 locomotor, 7 manipulative and 4 balance/stability). Within those, a variety of play or movement forms were also incorporated; for instance, pretend play (e.g., pretending to jump over a river, walk on a log or move like an animal) and movement to music. The workshop was embedded with an emphasis on: a) enhancing confidence and motivation through messages and demonstrations about challenge and adaptations; b) enhancing knowledge and motivation through role modeling, co-activity and following the child’s interest (choice); and c) education about how each activity influenced physical literacy inclusive of all physical literacy domains [6–8]. Play and play-based learning [43] – helping parents to create a joyful understanding of movement and play within their children was at the core of the workshop. This has been described more generally as tacit or experiential knowledge [44, 45] or, specific to physical literacy, categorized as comprehension of movement [46]. A play-based approach reflects pre-school children’s developmental stage and recommendations for physical literacy interventions to incorporate opportunities for pre-school children to engage in play [47]. The role of parents was both to facilitate and co-participate in active play rather than engaging in more structured motor skill development and teaching of codified knowledge that occurs in education and sport settings [23]. Other physical literacy concepts were delivered within and between activities through key parent messages which are identified following with their related child-level physical literacy components and domains (PLC/D):

- Fun, playfulness and play motivates children’s engagement (*PLC/D: motivation/affect*).
- Choice is important; follow your child’s interests where possible and let them choose the level of challenge they are comfortable with. (*PLC/D: motivation and confidence/affect*).
- Use a variety of activities and create many opportunities and an ‘invitation’ for children to move. (*PLC/D: motivation/affect*).
  - ◦ Manipulative skills are important to ongoing engagement in moderate, vigorous, physical activity and it is possible to do manipulative skills safely indoors with soft materials like balloons, scarves and paper balls and sticks. (*PLC/D: FMS/physical*).
- Many activities can be played both indoors and outdoors with inexpensive, accessible, simple home-made equipment. (*PLC/D: FMS/physical*).
- Avoid equipment or activities that may result in children getting hurt or developing negative associations (e.g., use soft materials such as scarves or balloons to practice catching). (*PLC/D: confidence and motivation/affect*).
- Start with small, achievable activities as initial success is important for children’s confidence.(*PLC/D: confidence/affect*).
- Modify activities to increase the level of ‘challenge’ where needed. This will facilitate continual development of skills and assist with motivation. (*PLC/D: confidence/affect*).
- Be a role model and be playful; children love to play with their parent(s) and this will assist to build the parent-child connection. (*PLC/D: motivation/affect*).
- Outdoor play is important; children move more in larger spaces and nature facilitates exploration, challenge and curiosity. (*PLC/D: FMS and motivation/physical and affect*).

### Quantitative data collection

Participants in the intervention group completed paper surveys in-person, immediately before and after their participation in the workshop. Participants in the control group completed online surveys a minimum of seven days apart prior to workshop attendance using a REDCap® [48] personalized link sent via email (Fig. 1). The outcome measures were chosen as indicators of parents’ capability, motivation and opportunity [10] targeted by the PLAYshop.

#### Parent characteristics

Baseline surveys collected parent characteristics including age, sex, level of education, number of children, and previous training in a similar topic. Additional questions explored parents’ modeling of physically active behaviors using three items from the Activity Support Scale for Multiple Groups (ACTS-MG) [49], and parents’ co-participation with their child using four items from the psychometrically validated physical activity parenting practices (PAPP) item bank [50].

#### Parents’ knowledge and confidence (study objective 1a)

The primary outcomes of interest were parents’ self-reported knowledge and confidence assessed at baseline and follow-up. Individual scale items focused on key physical literacy constructs derived from Canada’s Sport for Life [23] and PISE physical literacy experts (see Additional file 1). Knowledge was assessed via nine items measured on a 5-point Likert scale (1 = no knowledge to 5 = a lot of knowledge). Confidence was assessed via 11 items measured on a 5-point Likert scale (1 = no confidence to 5 = a lot of confidence). Cronbach alpha (α) tests at baseline and follow-up revealed acceptable values of reliability for knowledge (0.92 and 0.94) and for confidence (0.93 and 0.95) [51].

#### Mechanisms of change (study objective 3)

Parents’ beliefs, perceived barriers, outcome expectations and perceived availability of resources were assessed at baseline and follow-up using items, in their original format or modified for the parent perspective, from a previous instrument with high measures of internal consistency and test-retest reliability [52]. Each item was measured on a 5-point Likert scale from 1 = strongly disagree to 5 = strongly agree. Parents’ beliefs was assessed via four items (baseline α = 0.89; follow-up α = 0.81), perceived barriers via five items (baseline α = 0.50; follow-up α = 0.58); and outcome expectations via three items (baseline α = 0.86, follow-up α = 0.79). Perceived availability of resources focused on a unique PLAYshop target measured via a single item: Do you feel you have the resources (e.g., information, equipment, space, etc.) you need to promote physical activity and physical literacy for your children?; measured on a 5-point Likert scale from 1 = Yes, I have all of the resources I need to 5 = No, I don’t have the resources I need.

### Quantitative data analysis

SPSS Version 21.0 was used to analyze all quantitative data. Descriptive statistics were generated for all outcome measures and one-way ANOVA was used to determine if there were any significant differences between the groups in baseline characteristics. To address study objective 1a and 3, repeated measures ANOVA were used to determine if outcome variables changed over time across groups and if changes differed significantly between intervention and control groups (group-by-time intervention effect). Statistical significance was set a priori at *p* < 0.05. Effect sizes were reported as partial eta squared (η^2^) with a small, medium and large effect indicated by values of 0.01, 0.06 and 0.14 [53].

### Qualitative data collection

To address study objective 1b (changes in parenting practices) and study objective 2 (implementation facilitators and barriers), an experienced interviewer (MP or MK) conducted 5-10 min semi-structured telephone interviews. Parents from the intervention group were invited to partake in an interview two months following workshop attendance. Open-ended questions focused on their application of workshop learnings at-home, including what activities they had performed and what had made it difficult and/or easy to do so. For the study objective 2, workshop leaders were also interviewed in May 2020 to explore facilitators and barriers of workshop implementation and areas for improvement. All interviews were digitally recorded, transcribed verbatim, de-identified and uploaded into QSR NVivo [54] for thematic analysis.

### Qualitative data analysis

Qualitative data were inductively analyzed following the process recommended for multi-disciplinary health research [55]. Two members of the research team independently coded interviews and developed a working analytical framework. Where possible, data were charted into an NVivo [54] matrix to support interpretation of causes, consequences and relationships [56]. The research team discussed and reached negotiated consensus regarding any controversial codes or categorizations, and confirmed the proposed final themes. Concurrent with this process, parent interviews were deductively analyzed to explore the breadth of workshop elements applied at-home (frequencies reported).

Data authenticity was transparently affirmed through data display, exploring deviant cases and establishing trustworthiness of the findings using four quality concepts: credibility, transferability, dependability and confirmability [57]. Credibility and transferability were addressed through a rich and accurate description of the participants, context and setting. Credibility was further established through prolonged engagement of the research team with parents and in the delivery setting. Dependability and confirmability were addressed through the verification of transcript accuracy by interviewees, the use of triangulation across researchers during coding and interpretation, member checks with workshop leaders, and peer debriefing.

## Results

### Quantitative findings

**Fig. 2:**
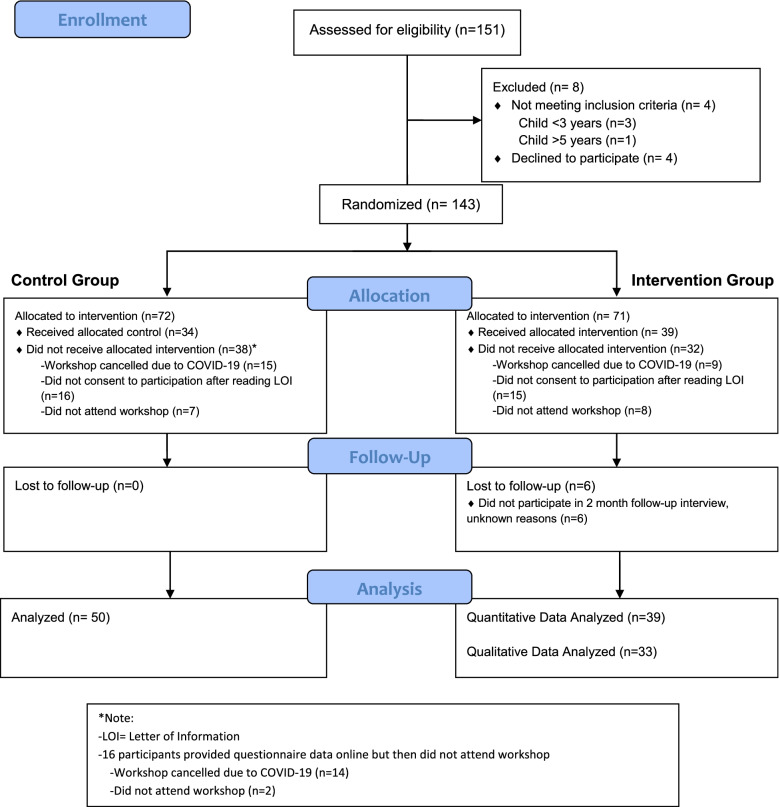
PRISMA flow diagram of participants’ progression through recruitment, group allocation, follow-up and analysis

Figure 2 details the progression of participants through recruitment, group allocation, follow- up and analysis. Of the 151 parents assessed for eligibility, four did not meet our criteria and four declined to participate. The remaining 143 parents were assigned to either the intervention group (*n* = 71) or control group (*n* = 72).

For the control group, 69% (*n* = 50) of parents provided survey data and 47% (*n* = 34) proceeded to take part in a workshop (68% of survey respondents). For the intervention group, 55% of parents (*n* = 39) provided survey data; all of whom participated in a workshop and 33 (46%) of whom participated in the two-month follow-up interview (i.e., complete quantitative and qualitative data). Failure to receive the workshop was the result of disinterest following reading the letter of information (LOI), no-show, or COVID-19 cancellations. Of note, interested families were allocated to a group when sent the LOI because the LOI was different for the intervention group and control group. These families (intervention *n* = 15; control *n* = 16) who failed to receive the workshop never consented to participate. A total of 89 (62%) parents provided complete survey data across groups (average age 36.1 years; 93% female; 14% with prior training in physical literacy; 2.01 average children per household). The characteristics of parents at baseline were not significantly different between groups (Table 2).

**Table 2:** Characteristics of parents at baseline

	Assigned Group	
Characteristic	Control (***N*** = 50)	Intervention (***N*** = 38^a^)
Age		
• Mean (SD)	36.7 (6.19)	35.5 (5.18)
Sex		
• Female – n (%)	48 (96%)	34 (90%)
Education level		
• Mean (SD)	3.90 (0.86)	3.87 (1.02)
○ Less than high school diploma	0%	0%
○ High school diploma	6%	11%
○ College or trade cert. or diploma	24%	26%
○ Bachelor’s degree	44%	29%
○ University certificate, diploma or degree above Bachelor’s level	26%	34%
Physical literacy-related training		
• Total – n (%)	36 (72%)	27 (71%)
○ Physical activity	26%	24%
○ Physical literacy	12%	16%
○ Fundamental movement skills	14%	13%
○ Sedentary behaviors	10%	5%
○ Other	10%	13%
Number of children		
• Mean (SD)	2.1 (0.83)	1.9 (0.69)
Modeling of physically active behaviors	*N = 49*	*N = 39*
• Mean (SD)	8.7 (1.93)	9.0 (2.05)
Co-participation with child in physical activity	*N = 50*	*N = 39*
• Mean (SD)	12.7 (3.93)	13.6 (3.85)

^a^One participant in the intervention group did not complete the characteristics section of the baseline survey

#### Parents’ knowledge and confidence (study objective 1a)

Table 3 shows the group-by-time significant intervention effects found for parents’ self-reported knowledge and confidence (*p* < 0.001) represented by large effect sizes (ɳ^2^ = .32). Parents’ self-reported knowledge and confidence improved significantly from baseline to follow-up in both the intervention and control group; however, the changes in the intervention group were significantly greater than those in the control group (data not shown).

**Table 3:** Results from the repeated measures ANOVA for parents’ self-reported quantitative outcome measures (group-by-time interaction)

Parents’ self-reported outcome measures	Group	N	Baseline mean (SD)	Follow-up mean (SD)	F	p	ɳ^2^
Primary outcomes (study objective 1a)							
Knowledge** *(max score = 45)*	Interv.	34	28.32 (6.27)	37.06 (4.73)	32.21	< 0.001	.32
	Control	39	29.03 (6.10)	30.18 (5.38)			
Confidence** *(max score = 55)*	Interv.	33	37.36 (8.0)	45.78 (5.69)	31.99	< 0.001	.32
	Control	38	39.34 (7.54)	38.82 (6.95)			
Mechanisms of impact (study objective 3)							
Beliefs *(max score = 20)*	Interv.	33	18.48 (2.0)	19.45 (1.33)	.834	.364	.01
	Control	40	18.25 (2.92)	18.68 (1.75)			
Perceived barriers* *(max score = 25)*	Interv.	33	11.49 (3.47)	14.55 (4.48)	4.86	.031	.07
	Control	32	11.75 (2.72)	17.50 (3.93)			
Outcome expectations *(max score = 15)*	Interv.	35	13.50 (1.40)	14.10 (1.25)	1.04	.312	.01
	Control	42	13.24 (2.03)	13.33 (1.43)			
Perceived availability of resources**^a^ *(max score = 5)*	Interv.	36	3.08 (0.73)	1.94 (0.96)	18.72	< 0.001	.19
	Control	42	2.81 (0.77)	2.62 (0.76)			

**Statistically significant (*p* < 0.001)

*Statistically significant (*p* < 0.05)

^a^A reduced score reflects improvement

#### Mechanisms of change (study objective 3)

Significant group-by-time intervention effects, represented by medium-large effect sizes, were also found for parents’ perceived barriers (*p* = .031; ɳ^2^ = .07) and perceived availability of resources (*p* < 0.001; ɳ^2^ = .19), but not for beliefs (*p* = .364; ɳ^2^ = .01) and outcome expectations (*p* = .312; ɳ^2^ = .01) (Table 3). Scores for parents’ perceived barriers, perceived availability of resources, self-reported beliefs, and outcome expectations improved from baseline to follow-up in both the intervention and control group. These changes in the intervention group were significantly greater for all measures with the exception of outcome expectations (data not shown).

### Qualitative findings

Thirty-three parents from the intervention group participated in an interview; 26 of whom verified their interview transcript. All five workshop leaders participated in an interview and verified their transcript.

#### Parenting practices (study objective 1b)

Parents reported a variety of workshop activities performed at-home with their child (see Table 4). The most common was manipulative skill activities, with 26 parents reporting at least one activity that fell within this category, followed by activities related to locomotor skills (*n* = 13), balance/stability/creativity (*n* = 11), and multiple-skills at one time (*n* = 9; for example, an activity that involved both balance and jumping). All interviewed parents stated that they were likely to continue performing physical literacy activities at-home with their child.

**Table 4:** Parent-reported physical literacy activities performed at-home, with sample quotes (study objective 1b)

**Manipulative skill games**	V114: Yeah, we’ve played with some of the materials; the tennis racquets with tape and nylon, and the scarf, and the ball. And then we do get outside every day to do some sort of soccer or practicing baseball.
	E205: Well one of our favorite is the one with the beany-bags … I have a basket and they can just throw it in there … and we do the one you showed us with the bottles and then tossing a ball. So instead of bottles we’re using blocks; we make the tower first and then tossing the ball, so it’s like double skills.
	E222: … using the little paddles to try to hit balloons back and forth and the bean bags, tossing them into a laundry basket and bouncing the ball kind of off the ground into the basket.
**Locomotor skill games**	V105: Riding the bikes actually, they absolutely love riding bikes.
	E200: Lots of just different running games or hopping games.
	E220: … he likes the one with the little scarf where we kind of play tag and you pull the scarf.
**Balance/stability/creative skill games**	V113: We worked on balance … walking on a little rock wall as we walk … in the neighborhood, he might balance on that.
	E213: … we’ve been having dance parties more often.
	E214: We’ve been catching the scarf on various body parts and having [my son’s] younger brother play with us and catch the scarf.
**Multi-skill games**	V113: … made it into little obstacle courses for him, and then sort of with chalk on the ground and got him to do different bits that way.
	E222: We have been doing a lot of obstacle courses but we kind of incorporated some of the things from the workshop … like the different types of jumps and walking on a straight line.
	E224: … we set up lots of obstacle courses for her to play with.

Parents also revealed their application of several key workshop messages at-home (see Table 5). For example, several parents cited that they had engaged in activities that were: fun/enjoyable experiences (*n* = 11), participated in whilst outdoors (*n* = 9), used minimal or inexpensive equipment (*n* = 13), child-led (*n* = 5) and challenging (*n* = 3). Ten parents mentioned that their child experienced additional benefits (beyond physical literacy skill development) by participating in workshop-related activities.

**Table 5:** Parents’ application of key workshop messages, with sample quotes (study objective 1b)

**Child-led play**	V114: I guess my daughter likes really just free style activities. For example, she’d be happy to just dance … she’s not so much into the, ‘this is what you’re supposed to do with this tennis racket’. She’s more into sort of playing with it and starting to goofing around with it.
	E213: What makes it easy? Well just that...I guess that she can do them on her own sometimes.
	E224: When she’s the one showing interest in the activity and letting her make the rules and letting her initiate the activities, that’s what’s making it very positive and successful over, ‘let’s do this’ or ‘let’s try this’.
**Outdoor play**	V113: We’re busy outside playing all the time.
	V116: Just being more active to play a little games and stuff outside, like throughout the day.
	E223: With all the COVID stuff we’ve been trying to just play outside as much as we can. She has a really awesome snow fort and a little toboggan run in the back yard that we just go up and down all the time.
**Minimal/Inexpensive Equipment**	E200: Well I think at the workshop it just made me realize that I didn’t need a lot of stuff. That it could just be just their bodies, or something really silly like a balloon.
	E205: Well the way that you showed us was with things that we have at home... so that has been really easy and sometimes because it is just things that are around the house, I don’t really have to prep. So, if we see something we can throw into a basket or wherever it’s just easy to do it.
	E207: It was nice to see different ways and different things we can use around the house or what we normally have at home to encourage this kind of physical literacy in her.
**Make it fun**	V116: We’ve played around with some of the games that you guys sent home, and that has been really fun.
	E202: He enjoys them, so he’s really engaged.
	E223: ... she’s just wanting to do [activities] because it’s fun, and it just makes her smile to play chase or to play tag or like the flag tag – we played that for a long time.
**Variety**	V110: My son loves doing an obstacle courses, so it’s usually about four or five different tasks – simple tasks. One thing was throwing the beanbag in the hoop. Another one is the racquets you provided us with; using those to bounce the ball. We have a slide in our backyard, so part of it is climbing up the play gym and going on the slide. Kicking a ball to the fence … a whole bunch of different things like that. And I just try to mix it up as much as possible.
	E200: We’ve been using hula hoops and the bean bags a lot. So throwing different things into that or taking things and throwing into a bin. Lots of just different running games or hopping games.
	E206: We have used a lot of the different activities … would do them for a while and then put the stuff away that you guys gave us … and then bring it back out again so it’s kind of like a refreshed look at everything … and then we’ll do different things in between.
**Challenge**	V105: In the garden, I’ve also been setting up challenges for the kids cause obviously with COVID-19, we haven’t been able to do lots of things in parks.
	V107: Yesterday we went tree climbing.
	E210: Since my kids are a little bit older … we put words on the wall and try to use the badminton racquets and balloons to touch different words and then say them.

#### Implementation barriers and facilitators (study objective 2)

##### Parent perspectives

Parents experienced numerous facilitators and/or barriers to implementing PLAYshop activities at-home (see Table 6). Themes that arose as facilitators included the simple and inclusive nature of activities, the minimal/inexpensive equipment needed to carry out these activities, and the ability to undertake activities without child enrolment in a special program. Themes that arose as barriers included busy schedules/lack of time, limited indoor space, younger siblings, sustainability difficulties, unfavorable weather, and the short attention span of some children. COVID-19 was mentioned in several interviews as either a facilitator or a barrier. For example, several parents indicated that the increased time spent at-home provided an opportunity to engage in PLAYshop activities (facilitator) whereas some parents felt the disruption to regular schedules made it challenging to facilitate active play opportunities (barrier).

**Table 6:** Parent-reported facilitators and barriers to implementing physical literacy activities at-home, with sample quotes (study objective 2)

**FACILITATORS**	**Minimal/inexpensive equipment**	V101: We’ve been building stuff out of random things instead of buying toys and stuff.
		E206: It doesn’t require spending money or a lot of props or things you have to use. And it allows me to be creative ... like what else do I have around the house that we can use as well?
		E207: I have all the stuff I need to do these. I have everything already at home, I don’t need to go out and buy anything and they seem to entertain her.
	**Simplicity**	V112: I’m more aware of how simple it is to just incorporate it into our daily activity.
		E211: … [the kids] are interested in it even though it’s such a simple thing … it doesn’t have to be really extravagant for them to enjoy it.
		E213: I really enjoyed the workshop and I thought it was a great idea, and just to learn these strategies and getting moving is not as difficult as one may think it is.
	**Do not need to attend programs**	E205: Sometimes you can get stuck in thinking, “I can’t do much at home” or “I have to go to a gym or rec center to do these kind of things”. But the workshop just kinda opened my eyes for other actives that we can do at home very easily and that kind of inspired me to find other similar things that we can do.
		E208: I think actually like the workshop was really eye opening in terms of letting me see the simpler things that actually were building my kids physical literacy. Whereas, before I think I thought they had to be in soccer to be building [physical literacy].
		E223: It’s not like you have to enroll your kid in every soccer class or enroll your kid in every [multi-sport program] or whatever the latest trend is. You don’t need to do that, you just need to have fun.
	**Inclusive**	E204: If one person picked it up, then the other person would join in and it was very inclusive.
		E205: I also have a 22-month-old boy … I find it’s hard for him to be included in other kind of games but … these more active kind of games are better for him as well.
		E206: Even my 9-year-old does them and thinks it’s fun.
	**COVID-19 (facilitator)**	V100: While he was in school I did not really do much at home, but with distance learning I was more motivated to do it and learned a lot so I think I would like to continue even during summer and when my son goes back to school.
		V114: It’s nice to be able to try new things, mix it up a little bit and just have some new ideas to do, especially right now when we’re at home for so much of the day.
		E209: … a lot of the games that they showed us in the workshop ...we’ve been doing variations of that since we’re quarantined in the house.
**BARRIERS**	**COVID-19** **(barrier)**	V119: Our world has been kind of a bit different in the last few months, and our routines have been off a little bit, but I think when things normalize a little bit and get into a bit of a different routine we’ll do some more of the other activities.
		E223: … balancing with all the work from home stuff happening right now – I think that’s the biggest challenge.
	**Busy schedule/lack of time**	E206: … me taking the time to get them going and doing it – that’s probably the biggest challenge.
		E211: … with busy schedules … really actually trying to carve out time to do activities like this for more than five minutes here, five minutes there.
		E215: … other barriers to doing the activities would be while you’re working it’s just busy-ness … you need to get meals going and [the activities] do include a lot of interaction.
	**Limited indoor space**	V107: Well we have a fairly small space so bouncing the balloon around, we do that, but I prefer we do it outside.
		E214: We got kind of a busier, tighter space in the house. So some of [the workshop activities] are hard to do and our back yard is basically inaccessible during the winter.
		E218: … space concerns ‘cause I live in an apartment and it’s really congested.
	**Child’s attention span**	E207: What makes it hard? I guess just her attention span.
		E220: … the only thing is his attention span is maybe a little bit short. So sometimes I’ll set up something and it takes a while to set up and then he’ll play with it for like five minutes … that’s maybe the challenging thing.
		E224: … a child’s attention span … if you wanted to do a specific fine motor or gross motor skill … but you only have a certain amount of time and there’s certain amount of attention span – it’s a little challenging.
	**Younger siblings**	E209: … the two little ones can’t obviously do [the workshop activities], so you have to wait until they’re busy before you can do [the activities] with the older one.
		E214: … his younger brother … it ramps him up and he’s not always totally physically aware of his space ... so it can make it hazardous to his [younger] brother depending on how it’s going.
		E222: I’ve had to put [the youngest child] in the jolly jumper just to stop her from messing up all of the obstacles.
	**Sustainability**	V100: Definitely less motivated for me when she is not engaged and interested at all …
		E201: I haven’t been doing them [the workshop activities] consistently enough, but they really enjoy them.
		E204: They haven’t lasted that long but they were interested in it for a little bit.
	**Weather**	E205: … once the weather is nicer I think we can just go outside and do [the workshop activities] and they have more area to do [the workshop activities].
		E224: … weather wise may be a little bit harder.

##### Workshop leader perspectives

Thematic analysis revealed several key facilitators and barriers to workshop implementation (see Table 7). Facilitators related to attributes of the workshop itself (starting with active play, working as a team with other leaders, the presence of strong/adaptable leaders, maintaining the tempo/flow, focusing on equipment that is easy to find and/or make, and providing parents with ‘goody bags’) or the workshop context (champion within the workshop site, engaged parents, children in attendance, and favorable physical spaces). Barriers were primarily related to the workshop context, including parents who were unengaged or felt shy to participate, issues with recruitment and low attendance rates, children’s disruptive or distracting behavior and parents struggling to manage this, unfavorable physical spaces (setting), and parking issues.

**Table 7:** Facilitators and barriers to workshop implementation from the perspective of workshop leaders, with sample quotes (study objective 2)

**FACILITATORS**	**Starting with play**	V1: We laid out equipment in preparation which is great ‘cause the kids engage early… they’re already vibrantly engaged. The families are thrilled cause they’re not trying to control their kids behavior
		V2: I think it was really important … to start engaging [the children] with play from the beginning, like showing them what to do so they felt like they were very comfortable in the room.
		E4: We also found it super beneficial to have lots of games for the kids to play at the beginning, while the parents are filling out consent forms or questionnaires.
	**Working as a team**	V2: I also think it really helped that we kind of worked as a team, like some [leaders] got the activities ready and some [leaders] talked with the parents, and some [leaders] were able to just introduce things.
		E5: I think for [Leader 4] and myself, we worked together quite well and understood each other’s roles quite well.
	**Strong/adaptable workshop leaders**	V1: Proactive leadership with energy … the modeling of the fun, and engagement, and energy is critical to leadership … it’s more modelling but it is also critical to engaging the kids and engaging the families.
		V2: We kind of just did it with a lot of energy and tried to figure out [differences] … cause some families and children are more engaged than others.
	**Tempo/flow**	V1: … the tempo of the workshop; the time between the different activities worked very well. Nobody really gets bored … they actually like the activity while you’re introducing another activity and then they might be more encouraged to do it at home.
		V3: I like the activities that were in there and the flow between the PLAYshop elements … it all went really smoothly together.
		E4: … when things started to get not as exciting, moving on quickly so that they weren’t getting bored and they were staying engaged.
	**‘Let’s make’ equipment**	V1: I think engaging the ‘Let’s Make’ stuff is critically important ‘cause we may not all have the resources to give them a goody-bag.
		V2: I think also the ‘recycled’ part works very well because … it’s more accessible to have these things at home.
	**Goody bags**	V1: [The parents] absolutely loved the handouts.
		V2: I think the goody-bag worked ‘cause they have some of the gear … they have the things to actually do some of the activities.
		E4: I thought that the goody bag that we gave them at the end was great, and a lot of them were really excited about the items in there.
	**Community champion**	V1: … having a really engaged community champion that actually helps with recruitment.
		V3: … having contacts with rec centers and daycare centers or whatever facility you’re working at that are really excited about the workshop makes it easier as well.
	**Organization**	V1: I think our planning did work … we had a worksheet of the schedule and the activities – I referred to it to prep every time. So, prepping every time using the tools I think really matters. So, early set-up, reminder of activities, dividing up activities across the staff if you’re sharing load, I think all those things helped.
		V3: … as long as you can do your set up quick and then you have all of your supplies ready for parents. We found that having all [parent’s] sheets labeled and enough clipboards made it really easy to get the workshop underway.
	**Engaged parents**	V2: … motivated parents was a huge help for the workshop, ‘cause they could encourage the children…
		V3: I think the parents being eager and excited about physical literacy and how they could get their children involved made it really easy to facilitate the workshops.
		E5: I think it was easier when the parents were more excited about it, and their children’s temperament was a little bit easier … if they were interested in, if their parents were interested, then it went well.
	**Having children attend**	V1: I liked that siblings could come ‘cause I think it helps with recruitment.
		V2: … some parents that showed up without their children cause they didn’t [think] that they could bring their children … maybe we should emphasize that.
	**Favorable spaces**	V1: We ended up with some good locations … we had very good spaces with enough room for movement and typically not shared.
		E5: We only had one gym or like one room. It was a multipurpose room, so it was a good size, like it was enough space.
**BARRIERS**	**Participants feeling shy/embarrassed**	V1: … we did have two parents come, they didn’t think they should bring their child and they left early cause they felt embarrassed ...
		V2: … one of them [the workshops] … sometimes the children and also the parents could be … kind of like feeling shy. Like, “should we just do it”?
	**Parking issues**	E4: … near the end we had some issues with the room we were renting; [the room] was in a rec center that was really busy … so parents were struggling to find parking, unfortunately.
		E5: There was a couple of weekends where the facility we were at … had all kinds of tournaments booked … we couldn’t actually park on the property. We had to park really far away. I think sometimes parents might be a little bit late because of that.
	**Recruitment & low attendance rates**	V1: Recruiting is the biggest barrier.
		V2: … the biggest problem within our delivery was probably the number of families showing up.
		E4: We had one workshop where one or two parents didn’t show up. So only one parent was there with their child.
	**Children’s behavior**	V1: … the younger children that were there that were on the [age range of three to five] or the siblings that were below the [age range] of three, caused some havoc.
		V2: … they were actually hungry … it was kind of sometimes maybe making them not concentrate or focus … it had an impact on the parents, that their children were kind of upset about being hungry.
	**Unengaged parents**	V2: … we also experienced that the parents maybe knew each other, and they’ll be talking on the outside [of the group] and then we kinda work more of a childcare system where they just came and hangout while we were doing some activities.
		E5: ...the parent was not really like helping out or in some cases they might be on their phones, they weren’t doing some of this stuff.
	**Parent’s not managing children**	V1: we had this one [parent] that had two kids in a more small space and she just let them engage with the equipment whenever they liked, however they liked. If they did something that wasn’t intended with the equipment it interfered with all the other children and made it a dangerous situation. We literally had to step in.
		E4: … one parent brought their one preschooler and two or three, school-aged children, and they just kind of took over the games and weren’t really listening. And the parent wasn’t asking them to listen ...
	**Children as a distraction**	E4: For a while we were playing a different game, but [the kids] were getting excited and kind of screaming. And so parents were not listening to me anymore. They were listening to the kids and watching the kids. I think they might’ve missed some of the messaging then.
		E5: … if the children wanted to hang out with their parents the whole time … they wouldn’t really want to come aside and play. They just want to stick to their parents. And there might be a little bit of distraction there.
	**Unfavorable spaces**	V1: Some of the spaces are noisier than others.
		V2: … I think some of [the spaces] were very small and some of them were very big, and when they were very big we usually split it, like made it smaller, ‘cause I think one time we had not a lot of children in a really huge space and that was very difficult cause then they’re all over the place.
		V3: In the [Victoria space B] one specifically … it was also very, very big. So it was hard, if parents were too far away, to hear us.

## Discussion

Leading organizations such as the WHO [13] have suggested that physical activity promoters should engage parents in their child’s physical literacy journey, however, few evidence-based programs were available to assist this. The multi-strategy PLAYshop intervention provides one such option, with high ratings of acceptability and suggested efficacy [34]. The current controlled trial expanded upon a prior feasibility study of the PLAYshop, using a more robust design, in a larger population and within an additional region. Compared to controls, significantly larger improvements for the intervention group were found in parents’ self-reported knowledge, confidence, perceived barriers and perceived availability of resources. Following the workshop, parents also reported engaging in workshop-related activities at-home with their child. Collectively, these findings establish the preliminary efficacy (measured at the parent-level) of the PLAYshop as a promising intervention option for the development of physical literacy in early childhood.

Our positive findings build on those established in the prior feasibility study [34]. However, the current trial explored an improved PLAYshop intervention with two new strategies (material resources and follow-up support) to support parent’s application of workshop learnings at-home. This change appeared to address the ‘lack of follow-up’ previously reported as a significant implementation challenge [34]. No parent expressed difficulty recollecting workshop learnings and/or implementing suggested activities at-home, and workshop leaders reported that the new strategies were indeed a prominent facilitator to implementation. However, several previously identified implementation challenges remained: recruitment issues in the Victoria region, unfavorable workshop spaces and children as a distraction despite the presence of a workshop co-leader. Further action to address these prior to scale-up should be considered and may be informed by investigating cross-regional experiences (e.g., the AB region did not have any issues with recruitment) and trials of similar physical activity interventions with reported success. For example, a recent feasibility trial of a lifestyle intervention for preschool aged children and their fathers [58] used a structured, multi-component, and targeted recruitment campaign that allowed the research team to exceed targets, achieving ≥60% recruitment of the eligible population. Similar to our workshop, the Healthy Dads Healthy Kids intervention also used a co-facilitator for parent-child dyad sessions to assist in the management of group activities [31]. The parent/child interaction varied across workshops and potential need for enhanced child management strategies should be planned for.

Our parent-level results are commensurate with that of previous parent-focused physical activity interventions targeting children’s FMS [32, 33, 58, 59]. However, children’s FMS was one of many targeted behaviors of these interventions, only two of which included FMS, one amidst several physical literacy components, as an outcome measure [58, 59]. Further, these interventions typically emphasized parents’ personal lifestyle behaviors and parent cognitive behavioral approaches was secondary. Evidence suggests that parent’s personal physical activity behaviors are less indicative of their support for children’s physical activity behaviors compared to social cognitive correlates such as their intentions, planning and perceived control [30] as well as encouragement, involvement and facilitation [60]. Theoretically, it was particularly important that parents in the PLAYshop were not only made more aware of how to develop their child’s physical literacy (physical capabilities, affective and cognitive elements), but also had enhanced knowledge and confidence to do so. This aim appeared to be achieved; parents demonstrated increased knowledge and confidence and the types of activities they performed at-home with their child were congruent with the workshop teachings. These are important findings in light of the significant relationship between parental self-efficacy and children’s physical activity [61].

Previous parent-focused physical activity interventions have also been more extensive, time-consuming, and/or resource intensive programming approaches. The PLAYshop offers a brief program focused on activities that can be performed at-home using easily accessible household items and requiring less of parent’s time in the program. Such characteristics are well-suited to address identified barriers of parents influencing preschool-aged children’s physical activity such as cost, opportunity, insufficient time and transportation [61]. However, it should be noted that while the workshop was ‘brief’ (accommodating for the barrier of time) and we supplemented with booster emails, there were still recruitment challenges in one jurisdiction and time remained a barrier to implementation in the home. With competing demands for attention in busy family lives (e.g., parent work commitments, travel, and having more than 1 child [62]) dedicating time for active play, regardless of how simple, remained a challenge. In our study, parents in the intervention group significantly improved in scores for perceived barriers and perceived availability of resources compared to controls. This is consistent with our qualitative findings that parent’s application of workshop learnings was facilitated by the minimal/inexpensive equipment required, simplicity of activities, and ability to perform activities at-home. The PLAYshop may effectively enable parents to overcome barriers and access resources to successfully engage in suggested activities. The potential of the PLAYshop to address multiple barriers may be particularly valuable seeing as the quantity of barriers experienced by parents has been found to be inversely proportional to the physical activity levels of children [61].

We found no significant intervention effect for parents’ beliefs or outcome expectations. This may reflect a ceiling effect: the mean baseline scores were no more than 1.75 below the maximum possible score for both of these measures (20 for beliefs and 15 for outcome expectations). Similarly, a pilot randomized controlled trial of the 8-week Mothers and Daughters Exercising for Life program found no intervention effect for mothers’ beliefs or outcome expectations, with negligible changes from baseline to follow-up [33]. This may be attributable to recruitment bias, at least in our study (see limitations following), however it is likely that the positive outcomes of physical activity are generally known by parents. In a cross-sectional survey of 663 Canadian mothers, 58% ranked physical activity as the first or second most important activity for their child - slightly higher than homework and far higher than other activities (e.g., music/arts = 14%; peer socializing = 13%, and family time = 39%) [63]. Another cross-sectional survey of 483 Canadian parents showed that 79% believed ‘health’ was an important reason to engage with their child in physically active behaviors [64]. Parents’ beliefs and outcome expectations may have less impact driving behavior change compared to other theoretical mechanisms of change, although further research is needed in this regards.

A unique aspect of this study was the workshop timing allowing us to explore the impact of COVID-19 lockdown restrictions during the study. COVID-19 was reported by several parents as either a facilitator (more time spent at-home and increased motivation) or a barrier (disturbed routine and difficulty balancing lifestyle changes) to their application of workshop learnings at-home. These findings complement a cross-sectional study by Moore and colleagues [65] that used an online survey of 1472 Canadian parents to explore the immediate impact of COVID-19 restrictions on movement and play behaviors in Canadian children and youth (5-17 years). The majority of parents reported their child as being ‘a lot less’ active due to COVID-19 restrictions, with significant declines in children’s time spent in physical activity, play and sport. However, several parents also reported adopting new hobbies or accessing new resources. Further analysis showed that parental encouragement, support, and engagement were positively associated with increased physical activity [65]. The PLAYshop successfully improved parent’s knowledge and confidence to engage in play with their child and emphasized the use of accessible household items – this appeared to be of substantial benefit under COVID-19 restrictions.

### Limitations

There are several study limitations that warrant acknowledgement. The AB region chose a systematic approach for group assignment to address challenges that were anticipated with the immediate scheduling of both intervention and control workshops in this jurisdiction. Although randomization is considered the gold standard for group allocation in controlled research trials, it is not always practical for public health research [37]. Another limitation was the retrospective registration of the trial after the date of the last workshop which ended unexpectedly due to the COVID-19 pandemic. However, no data was analyzed prior to trial registration. Further limitations include the following: (i) the research team was not blinded to group allocation due to their involvement with intervention delivery and data collection; (ii) fathers were underrepresented (93% of survey respondents were female) with evaluations most often completed by a female partner despite attendance by some fathers; (iii) the use of self-report surveys may be subject to social desirability bias, especially since those in the intervention group were completed with research staff available nearby (however this did provide the opportunity for parents to seek assistance if any survey question was unclear); (iv) the surveys were expert-driven but not sufficiently validated; (v) there was a limited review and feedback from a more diverse range of physical literacy experts (e.g., researchers and/or physical literacy groups in other regions) which may have improved content validity; (vi) the α scores of perceived barriers were in the lower range for reliability however still considered ‘fair’ [51]; (vii) there is a possibility of selection bias due to self-recruitment of participants as parents in this study typically agreed that they enjoyed physical activity and thus may differ significantly from those who did not choose to participate; and finally, (viii) the apriori sample size was based on an estimate, although post-hoc power analysis and the significant small to medium size effects detected indicate that the actual sample size was adequate and allowed us to detect differences between the groups. Our effect sizes were similar to a previous physical activity parenting practices study which reported small to medium effect sizes for measures of parents’ socio-cognitive measures, ranging from a Cohen’s d of 0.24 for support to − 0.45 for self-efficacy [33]. It is possible that the PLAYshop may have attracted parents who were already inclined to engage in active play with their child.

### Future directions and recommendations

This trial forms the second in a series of studies to optimize the PLAYshop for dissemination – that is, to develop the most effective and resource-efficient option [66]. Through this process, findings from the current study are used to inform improvements to both the intervention and to research processes, including addressing limitations where possible. For example, resolving insufficient representation of fathers in childhood health and physical activity research has been flagged as a research priority [35, 67]. Future research should incorporate father-specific recruitment strategies and methods to improve their response rates. Similarly, targeting those parents less engaged in physical activity will ensure a more representative evaluation sample. There are also opportunities to improve data collection methods (e.g., validating the surveys would improve the rigor of results) and to increase parent’s exposure to messaging and activity ideas with additional follow-up support strategies (e.g., a smartphone app).

The current trial was conducted largely in a controlled research setting with the research team driving recruitment and workshop delivery. Future research should be conducted in more ‘real-world’ conditions to test the effectiveness of the PLAYshop [68]. This could be achieved by using a train-the-trainer model and/or engaging external delivery partners to extend intervention reach and the delivery context beyond the research team. This would also provide implementation infrastructure support which is considered essential for successful scale-up [40]. Moreover, in light of the recent COVID-19 pandemic it will be important to test other scalable workshop formats (e.g., online/web-based) that are under development and less likely to be impacted by public health restrictions. This will also provide those parents who were unable to receive the workshop in the current study with an opportunity to participate.

The current study is one of very few targeting parents as gatekeepers for physical literacy, and the first of its kind to use a brief and feasible workshop to improve parents’ knowledge and confidence to do so. Therefore, this study provides a valuable contribution to an emerging field of work; however, several key empirical questions remain unaddressed, and should be a future priority in parent-focused physical literacy research. For example, whether parent’s physical literacy parenting practices impact on their child’s physical activity and physical literacy (objectively measured). It is also important to explore the impact of socio-economic status and ethnicity as these factors may significantly influence home life and the opportunities that children are provided by parents to develop physical literacy.

## Conclusion

We established that a 75-min, co-facilitated workshop with parent resources and follow-up boosters was effective in improving parents’ physical literacy-related knowledge and confidence. The workshop also appeared to meet other intervention targets – with a positive impact on parents’ perceived barriers, perceived availability of resources, self-reported parenting practices, and participation in suggested activities at-home. This study provides a valuable contribution for an emerging field of work and may be used to inform future research, policy and practice. Its findings are pivotal for optimizing the PLAYshop for future evaluation and scale-up.

## Supplementary Information

**Additional file 1.** The survey scales used to assess parents’ knowledge and confidence.

## Data Availability

The datasets used and/or analysed during the current study are available from the corresponding author on reasonable request.

## Abbreviations

FMS: Fundamental movement skills
WHO: World Health Organization
AB: Alberta
BC: British Columbia
PISE: Pacific Institute of Sport Excellence
ACTS-MG: Activity Support Scale for Multiple Groups
PAPP: Physical activity parenting practices
