# Supplementary material for: Exploring a parent-focused physical literacy intervention for early childhood: a pragmatic controlled trial of the PLAYshop
Source: BMC Public Health. 2022 Apr 5;22:659. doi: 10.1186/s12889-022-13048-5 (PMC8982907; doi:10.1186/s12889-022-13048-5)
Supplement: Supplementary file 1 — Additional file 1. The survey scales used to assess parents’ knowledge and confidence. [file 12889_2022_13048_MOESM1_ESM.docx]

Additional File 1 – The survey scales used to assess parents’ knowledge and confidence

**Circle the statement that best describes your KNOWLEDGE about the following areas regarding your preschool child.**

|  | No knowledge | Very little knowledge | Some knowledge | Quite a bit of knowledge | A lot of knowledge |
| --- | --- | --- | --- | --- | --- |
| Physical literacy | 1 | 2 | 3 | 4 | 5 |
| Locomotor skills | 1 | 2 | 3 | 4 | 5 |
| Manipulative skills (catching, hitting, striking, kicking, throwing) | 1 | 2 | 3 | 4 | 5 |
| Balance and stability activities | 1 | 2 | 3 | 4 | 5 |
| Moderate to vigorous physical activity | 1 | 2 | 3 | 4 | 5 |
| Facilitate active play | 1 | 2 | 3 | 4 | 5 |
| Creating a home environment that encourages active play | 1 | 2 | 3 | 4 | 5 |
| Adapting physical activities for my child’s abilities | 1 | 2 | 3 | 4 | 5 |
| Limiting sedentary behaviours (e.g. screens, prolonged sitting) | 1 | 2 | 3 | 4 | 5 |

**If I wanted to, I am CONFIDENT in my ability to provide activities to my preschool child that promote / include:**

|  | No confidence | Very little confidence | Some confidence | Quite a bit of confidence | A lot of confidence |
| --- | --- | --- | --- | --- | --- |
| Physical Literacy | 1 | 2 | 3 | 4 | 5 |
| Locomotor skills | 1 | 2 | 3 | 4 | 5 |
| Manipulative skills (e.g. catching, hitting, throwing) | 1 | 2 | 3 | 4 | 5 |
| Balance and stability activities | 1 | 2 | 3 | 4 | 5 |
| Moderate to vigorous physical activity | 1 | 2 | 3 | 4 | 5 |

**If I wanted to, I am CONFIDENT in my ability to:**

|  | No confidence | Very little confidence | Some confidence | Quite a bit of confidence | A lot of confidence |
| --- | --- | --- | --- | --- | --- |
| Model/participate in active play with my child | 1 | 2 | 3 | 4 | 5 |
| Adapt physical activities to my child’s abilities | 1 | 2 | 3 | 4 | 5 |
| Facilitate active play | 1 | 2 | 3 | 4 | 5 |
| Create a home environment that encourages active play | 1 | 2 | 3 | 4 | 5 |
| Limit sedentary behaviours (e.g. screen time, prolonged sitting) | 1 | 2 | 3 | 4 | 5 |
| Motivate active play | 1 | 2 | 3 | 4 | 5 |
|  |  |  |  |  |  |
